# An Entropy-Based Design Evaluation Model for Architectural Competitions through Multiple Factors

**DOI:** 10.3390/e21111064

**Published:** 2019-10-30

**Authors:** Orkan Zeynel Güzelci, Sinan Mert Şener

**Affiliations:** 1 Department of Interior Architecture and Environmental Design, İstanbul Kültür University, İstanbul 34158, Turkey; 2 Department of Architecture, İstanbul Technical University, İstanbul 34367, Turkey; mert@itu.edu.tr

**Keywords:** entropy, information theory, multi-objective genetic algorithm, non-dominant sorting algorithm, municipality building, architectural competition

## Abstract

Generally, the evaluations in architectural competitions are based on quality where many criteria are involved. Additionally, many other inter-related criteria, identified by the members of the jury, emerge during jury evaluation. Hence, a great number of criteria play a role, with varying degrees of importance, in the evaluation process. The order of importance and weights of criteria (factors) in the evaluation phases are not fixed and differ according to the approaches of the jury members. The objective of this study is to investigate whether subjective means of evaluation can be associated with an objective and computable evaluation model. Entropy, an objective method used to measure disorder in buildings, offers significant potential in enhancing the comprehensibility of subjective tendencies in jury evaluation of architectural competitions. Previous studies have identified an inverted U relationship between entropy and subjective responses based on single and multiple factors. The Entropy-Based Design Evaluation Model (EBDEM), a method, analyzes the level of objectivity in jury evaluation and questions the predictability of evaluations through examining the relationship between the entropy values of projects and success outcomes. The Weighted Overall Entropy (WOE) was obtained by multiplying multiple factor entropy values with different weight coefficients with the purpose of ranking each project on an inverted U graph similar to jury results. The relationship between WOE values calculated and the ranking of the projects in the competitions were investigated. The findings within this study indicate that there are no relationships between single factor entropy values and ranking of the projects. Additionally, it was found that WOE values calculated for single-competition compared to multiple-competitions were more similar to jury evaluation results.

## 1. Introduction

Since psychology started getting recognized as a scientific discipline, psychologists aimed to associate the behavior of people with the properties of their surrounding objects. Psychologists have analyzed how human behavior changes with features of stimuli such as complexity, uncertainty, disorder, ambiguity, and novelty. During these investigations, psychologists have benefitted from the approaches of measurement in information theory propounded by Shannon in 1948 [1]. As a result of Shannon’s information theory providing the ability to measure the disorder of objects (stimuli), researchers have been encouraged to seek new relationships between psychology and information theory [2,3,4,5,6]. Berlyne’s article [7], “Uncertainty and Conflict: A Point of Contact Between Information-Theory and Behavior-Theory Concepts”, provides reference directly to the investigated relationship.

According to Vitz [8] organisms can be considered as “information processing systems”. Considering this perspective, upon acquiring a level of information of stimuli, humans create their own judgements and responses to the information received. Berlyne [2,3,4,5] is a pioneer in such studies on the relationships between the physical properties of stimuli to psychological responses and subjective judgements. According to Berlyne [2], arousal arises from the presence of features such as complexity, uncertainty, and disorder of the stimuli. Information theory and relevant discussions address the measurability of aspects such as complexity, disorder, and uncertainty. These features can be measured through the use of entropy of which can be applied to both abstract and concrete materials such as sounds [9], visuals [10], and written [11] content.

Amongst mankind’s most important creations, cities and buildings have a high level of organized complexity and each can be handled as a source of information. People, in turn, process the information originating from information sources (buildings) through the filter of their own perceptive and cognitive tendencies. Differentiation of perceptive and cognitive tendencies renders it impossible for each person to make the same assessment. Various types of information embedded in buildings or built environments can be measured and rendered intelligible by using methods such as entropy to give objective, quantitative, and precise results. Entropy measurements can be conducted on built environments through factors such as size, scale, color, texture, shape, silhouette, and other non-ambiguous characteristics. In this field, a variety of experts focus on the entropy measurements of buildings and built environments [12,13,14,15,16,17].

The built environments in which human beings’ dwell in may cause responses and behaviors such as “aesthetic responses” and “aesthetic preferences”. Furthermore, the perceived quality of the urban surrounding is defined as “aesthetics” [4,18,19]. Investigating the connection between different design components and aesthetics are carried out by environmental psychologists. According to Berlyne [2], aesthetic preference (positive affect) is related to the level of arousal created by the physical properties of stimuli. Later, Berlyne [4] analyzed the relationship between arousal arising from perceived information content such as physical complexity and aesthetic preference.

Humans assess the aesthetics of their surrounding environment. Analyzing relationships between people’s feelings and various physical design components are referred to as “design review” [20]. Design review and environmental psychology studies are linked because design reviews are related to how the physical environment affects human preferences. Judgements based on how physical features influence the perceived quality of the building and its surroundings are conducted by design reviewers [18].

Based on this point, architectural competitions can be described as a unique field in which design review processes are conducted intensively to select the best project among many alternatives. Generally, the evaluations in competitions are based on the quality of projects. In this context, there is no set definition for the concept of quality, and also there are significant debates on how quality is evaluated and convey new meanings in different contexts. Many studies focusing on the review processes of architectural competitions seek answers to the following questions:

- What is the definition of architectural quality?
- How is quality assessed in a competition?
- How does the jury choose the winning project among many proposals?
- How does the jury choose the best solution?
- How are architectural competition projects evaluated?
- What are the main criteria for selecting winning projects?
- What are the evaluation phases of the award-winning projects? [21,22,23,24,25].

In architectural competitions, the designers try to propose satisfactory design alternatives considering many design criteria (factors) together as input [22,23,26,27]. Since there are multiple design problems addressed in the competition brief, the design criteria of the participants also have an open-ended character. While one criterion becomes essential in the design of one project, another criterion may be important for other projects. Since there are different strategies and complex relationships to be followed in the solution of the design problem, weights between design criteria become crucial [22,26].

On the other hand, during the evaluation of the projects that participated in the competition, the jury members use their own criteria which are not written in the competition brief. These criteria depend on expertise and knowledge of the design reviewer and generally correspond to the architectural quality [28]. While it is the jury choosing the prize-winning projects, the weight of the selection criteria may change, and new criteria may emerge during the evaluation process [29].

In previous design review studies, a single building or built environment is evaluated based on many survey respondents. Alternatively, in architectural competitions, a large number of projects are evaluated collectively by a number of jury members. During evaluation processes, due to the interaction of the selected jury, factors which have not been studied previously in literature may arise. New factors and their effects on preference (degree of importance) may also emerge during the jury’s evaluation process. With these features, the evaluation processes of the architectural competitions differ from design review approaches developed in scientific studies. In the process where all design review criteria are clear, the proposed designs could be checked and evaluated for each criterion precisely. However, in reality, the criteria are not as clear and precise; personal judgement plays a role in both individual and collective decision-making processes.

It is important to ensure fairness in the evaluation process of the competitive environment, which is one of the fundamental characteristics of competitions. Although, the staging of competitions and the selection of jury members in a way that they will evaluate the projects in an unbiased manner are actions taken to ensure fairness in architectural competitions. Architectural competitions try to set a competitive environment in which participants with different levels of experience can compete under equal conditions [21,30,31]. In spite of all the conditions that aim to bring objectivity to the forefront during the competition process, the jury’s evaluation of the projects over the qualities that are difficult to define adds a subjective perspective to the process. Of significance is the problem of fairly and objectively evaluating buildings through subjective approaches, such as jury evaluations based on personal taste and preference.

### Aim and Scope

The main objective of this study is to investigate the relationship between jury evaluation results of competition projects and measured entropy values of competition projects. The justification of our study is that there are no previous studies examining the relationship between the objectively measurable features of architectural competition projects and the design evaluation conducted by the jury. Thus, a new approach was developed with the help of the existing studies linking psychology, architecture and information theory. In this context, entropy, which is an objective method also offers significant potential in enhancing the comprehensibility of subjective tendencies that involve uncertainty during the evaluation of projects in architectural competitions as well as in better analyzing and questioning such tendencies.

Briefly, some detail regarding this study can be discussed. In addition to subjectivity affecting the jury evaluation process in the assessment of buildings, the order of importance and weights of criteria (factors) in evaluation phases are not fixed and differ according to the approaches of the jury members. Complex building designs involve numerous factors such as shape, height, scale, color, silhouette, and materials. Upon analyzing complex buildings, one requires a method to measure multiple factors and incorporate the significance of their relationships simultaneously. A proposed solution to these problems is a combination of multi-objective genetic algorithms and entropy calculation methods. The majority of the entropy measurement methods within the study were reinterpreted, while some entropy measurement methods were developed first-hand.

The principal conclusions intended to be pursued within this study are as follows:

- Can the result of subjective jury evaluations be predicted on the basis of the projects’ objective entropy values?
- Can subjective means of evaluation, like jury assessment, be associated with an objective and computable evaluation model like entropy?

Based on this point, the Entropy-Based Design Evaluation Model (EBDEM) was developed which aims to investigate the relationship between the multiple entropy values obtained by the objective measurement of competition projects designed and success achieved as a result of the subjective evaluation of an architectural competition jury. The expected results of this study are as follows:

- To understand whether there are optimum entropy values for certain architectural programs such as municipal buildings through architectural competitions.
- To gain insight on different factors and their weights through assessing many different projects and factors; to understand whether some factors are more dominant over others in the evaluation process.

In the following sections,

- Section 2 details the definitions related to the concept of entropy, then provides a solution to the problems stated within the introduction section and is also a keystone of the proposed model. Further, findings of the previous studies which investigate the relationships between subjective evaluations and entropy are examined (Section 2.1).
- In Section 3, the model (EBDEM) is discussed within the development process; parameters necessary to conduct entropy measurements such as architectural typology, projects, competitions, and factors to conduct entropy measurements are identified, as well as how the projects and their factors are represented for calculating (Section 3.1.1 and Section 3.1.2).
- For the selected factors in Section 3.1, reinterpreted and developed entropy calculation methods are explained in detail for each factor (Section 3.2).
- Section 3.3 introduces Weighted Overall Entropy (WOE) and its calculation method within the scope of this study; further, fitness functions to be used in the optimization of WOE values are identified and why Galapagos is an appropriate optimization tool is discussed.
- In Section 4, two experiments based on the application of EBDEM on municipal buildings are presented. The single factor entropy values of projects selected in Section 3.1 are calculated by referring to the methods explained in Section 3.2 (Section 4.1). Moreover, Section 4.2 calculates WOE values and weight coefficients simultaneously for all competitions. Section 4.3 provides findings based on the two experiments.
- Section 5 provides principal conclusions and answers to the expected results detailed within the introduction section. Lastly, Section 5.1 addresses further improvements in this study.

## 2. Entropy

Having originated in 19th-century physics (thermodynamics), the concept of entropy later interested researchers of biology [32,33], sociology [34], psychology [35,36], ecology [37], economics [38,39], art [40,41,42], geography [43,44,45], urban design [46,47], and architecture [14,15,16] and made its way into these disciplines [48,49,50,51]. While the main notions and concepts related to entropy were transferred between different disciplines, they have also been handled theoretically, used in quantitative measurements, or reinterpreted metaphorically.

In this study, the concept of information entropy used by Shannon in his information theory was used for the objective measurement of buildings. Entropy remained confined to the discipline of physics until 1948, when Claude Shannon rediscovered the concept of entropy, in information theory, to measure the disorder in information, as different from the approach to entropy Clausius and Boltzmann used in physics [1,51].

Shannon worked in the 1940s on communication problems and was the first to use the concepts of information and entropy in combination. Shannon introduced the concept of information entropy as a theoretical communication model and focused on digitization, representation, transference, and processing of information coming from a source [52].

According to Shannon,

- the quantity of information is associated with the predictability of the content of the message transmitted;
- the quantity of information that is carried with a message is dependent on the number of probabilities of outcome that can be created by that message;
- if there is only a single message transmitted continuously from the source and there is only one probable output, then there is no information transfer;
- entropy assumes its largest value if all messages have equal occurrence probability [50,52].

In information theory, the value of entropy can be calculated by taking the logarithm of the number of possible alternatives that can be transmitted via a message. The unit of the calculated information is called a “bit” as a condensation of “binary digit”, first introduced by John W. Tukey.

When the information source can create 16 alternative messages and the likelihood of these alternative messages being sent is equal; 16 messages are expressed as 2^4^ and according to the result of log_2_16 = 4, the entropy value of the source is calculated as 4 bits [53]. In the basic equation that can be used to calculate the entropy value; *H* denotes the entropy value calculated for a factor and is calculated as “bits”. Furthermore, *p* denotes the probability of the occurrence of a factor [14,17].

*n* level *H* = −∑ *pi* log_2_*pi* *i* = 1. (1)

Bailey [48] states that the concept of entropy that Shannon propounded can be applied to any information containing multiple different data types, regardless of context. For example; for a five-element string made of letters and for each unit to have four (*n* = 4) values, five letters are randomly created (A, B, C, D). In the ABCBD letter string, there is one count “A”, two counts “B”, one count of “C”, and one count of “D”. In this case, it is observed that the rate of frequency of the letters in the string is 1/5, 2/5, 1/5, and 1/5, respectively. Once the probabilities listed are inserted into the equation and summed, the overall entropy value of the letter string is calculated as *H* = 1.92 bits.

Eddington [54] was the first to use the concepts of entropy, beauty, and melody in a common framework; he argues that entropy is observable when there is unity among the pieces. Entropy, beauty, and melody co-exist via the association of visible or audible components. Weaver [53] emphasizes that entropy can be associated with the concepts of beauty and melody and is not solely dependent upon arithmetic. The ideas that Eddington had propounded 20 years prior to the development of the concept of entropy in information theory was utilized in later years with academic studies associating entropy and subjective responses. Considering these approaches, Section 2.1 investigates the studies in which entropy values calculated are associated with such subjective evaluations.

### 2.1. Relation between Entropy and Subjective Evaluation

Literature review reveals that researchers have used several different terms to refer to entropy and complexity. While Nasar [55], Elsheshtawy [56], and Stamps [57,58] simply use the term complexity, Wohlwill [59] uses diversity in lieu of complexity to avoid confusion with structural complexity. Kaplan and Kaplan [60], on the other hand, suggest the use of visual richness to avoid the negative connotation of the word complexity. Arnheim [41] points out that the concept of chaos issuing from complexity has come to be defined as disorder in scientific parlance. Similar to the concept of complexity, the concept of entropy has also been defined in several ways. The concept of entropy in information theory is used to measure the level of complexity, disorder, or uncertainty of a system [17,48]. While Berlyne [2] argues that entropy can be used to physically measure disorder, Cover and Thomas [61] define entropy as the measurement of average uncertainty. Shaw and Davis [62], on the other hand, have used entropy synonymous to the concepts of disorder and diversity. Evidently, the variety of definitions used by different researchers and studies of a similar nature might be measuring entropy, complexity, disorder, uncertainty, or diversity, depending on the term they choose.

**Figure 1:**
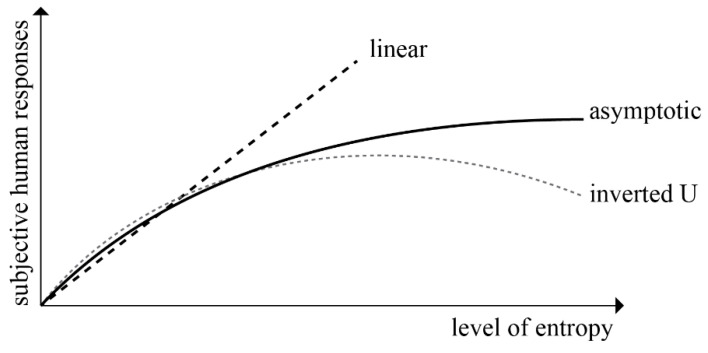
Three possible functions between subjective human responses and level of entropy.

There are many studies in the literature analyzing the relationship between the measurable complexity, disorder, diversity, uncertainty, and entropy amounts of various stimuli and subjective assessments based on personal like or preference. Berlyne [2,3,4] conducted the pioneering work investigating human responses to the complexity of concrete or abstract stimuli. Berlyne [2] theorized that positive subjective feedback was related to the level of stimulation created by the physical features of the stimulus. Stamps [14] argued that linear, asymptotic, quadratic (inverted U) relationships could be observed between subjective human responses and the level of entropy (Figure 1).

The level of complexity of information obtained from sources or the level of uncertainty contained in the information positively or negatively affects the evaluation of objects or buildings. According to Berlyne [2,5], a low or high level of complexity in stimuli is not preferred since it causes boredom or confusion. While a high level of disorder in the information coming from the information source causes incomprehensibility and chaos; low levels of disorder leads to monotonousness and vapidity. However, numerous studies have found that an average degree of disorder creates positive feedback from people, as well as pleasure [4,5,9,10]. Vitz [9] and Crozier [63] investigated the relationship between pleasantness and entropy through the sound sequences they produced and obtained an inverted U graph. Saklofske [10] questioned the relationship between attractiveness and complexity through 15 pictures of human figures and likewise obtained the inverted U graph in conclusion.

As the studies examining the relation between entropy and subjective evaluation focus on the measurable qualities of a wide array of stimuli, a similar approach can be adopted with respect to architectural products, which also have measurable qualities. Under the scope of this study, EBDEM aims to measure the entropy in buildings. A review has been conducted of the studies calculating, by means of various methods; the entropy of buildings and analyzing the relationship between the entropy values of buildings and subjective responses.

In terms of architecture; it is thought that an average level of complexity in building elements, buildings, building blocks, and cities creates the feeling of unity within variety [56,64]. Additionally, Tabacchi and Termini [65] emphasized that unity in variety is the cornerstone of human judgement on aesthetic. Herzog, Kaplan, and Kaplan [66], Wohlwill [59], Nasar [55], and Imamoglu [67] emphasize that an average amount of complexity is positively associated with the preferences of people. Wohlwill [68] questioned the relationship between preference and complexity through images of built environments and re-concluded with an inverted U graph. The hypothesis of this study shows similarities with the results of given references which emphasize that an average level of entropy creates positive response and evaluation.

## 3. Materials and Methods

In literature, various objective and subjective methods are used for the assessment of built environments, buildings, and designs. While objective methods focus on the features of the buildings that can be calculated, subjective methods evaluate buildings from the aesthetic perspective, based on personal likes and preferences.

Architectural competitions offer architects the chance to present their design ideas, propose innovative solutions to design problems, meanwhile be recognized. The design brief, while an important component of architectural competitions that outlines the general framework of the competition and guides the participants to some extent by laying down the rules, falls short of defining the complex relationships which the buildings contain. Ambiguities in the design brief, which fail to define well the design problems involved, cause participating architects to have to interpret the brief and develop their designs not only on the basis of the design brief but also their interpretations. Likewise, juries in architectural competitions do not confine themselves solely to the design brief and evaluate the project both objectively and subjectively. Considering the overall nature of architectural competitions, it is evident that while the brief introduces objectivity to the evaluation process, the evaluations of the members of the jury based on their personal experience and preferences add subjectivity. Projects that participated in competitions are assessed against many measurable and unmeasurable criteria and qualities they should have. The fact that the concept of “quality” is relative with no set definition causes jury members to adopt a subjective approach. Additionally, many other inter-related criteria, identified by members of the jury, emerge during jury evaluation. Hence, a great number of criteria play a role, with varying degrees of importance, in both the design and evaluation processes.

The purpose of this study is to investigate the relationship between jury ranking of competition projects and entropy values of the projects. First, we discussed the appropriateness of the entropy method by comparing it with the Analytic Hierarchy Process (AHP) evaluation method. Then, we explained the outcomes of this comparison and identified the shortcomings of previous entropy studies to develop the proposed method. 

AHP is a multi-criteria decision making (MCDM) method developed by Thomas Satty [69]. AHP is used in the solution of MCDM problems where multiple decision-makers or reviewers are involved in the evaluation process to select the best solution from a large number of alternatives. In the application of AHP, there are multiple decision criteria and the main operation in the decision-making phase is to give importance (weights) values to these criteria [70]. During the implementation of AHP, the complex MCDM processes are divided into subparts. Thus, AHP enables reviewers to organize MCDM and its criteria as a hierarchical structure. Lastly, reviewers make their decision based on defined hierarchy, criteria, weights [71,72].

Application of AHP in the field of architectural design provides an effective approach to evaluate designs collectively according to many weighted criteria and select the best design solution. When design reviewers are dealing with a complex design problem including multiple abstract and concrete criteria, hierarchical structuring ability of MCDM problems become the main characteristic that distinguishes the AHP from other evaluation methods [73,74].

The use of personal preferences and judgment in decision-making is an important issue. In the field of architecture, it is necessary to make reviews by using both qualitative (abstract) and quantitative (concrete) data together because both criteria simultaneously affect the design and decision-making process. The hierarchy between qualitative and quantitative data can be defined as the main selection criteria and sub-criteria. The use of AHP allows reviewers to define their decision-making mechanisms by considering their evaluations in different psychological situations. AHP method distinguishes with its ability to convert both objective and subjective data into the numbers [75]. In the AHP, first, the pairwise comparison is used to determine and sort the importance and weights of criteria. As a result of the comparison, it aims to determine the effects of each criterion on the overall evaluation process. Weights of the criteria may be changed according to the intention, experience, and knowledge of the reviewers. Finally, design reviewers can use determined criteria and weights to evaluate the design alternatives.

In this study, our data set is limited to the project panels and drawings on the panels. Although we have the jury reports of the architectural competitions, these reports do not reflect or clarify the selection criteria of the individual jury members. To use AHP for the evaluation of competition projects, surveys or interviews have to be done with the jury members to determine the criteria and their weights. In these cases, the projects submitted to the selected competition have been evaluated and it is not possible to bring all jury members together to identify their collective selection criteria and the weights of each criteria. Moreover, in such project panels, only the physical properties (data) of the project are presented, rather than abstract data such as conceptual backgrounds. 

In the case investigated in this study, entropy values are calculated based on the drawings of award-winning projects chosen as a result of the reviewers’ evaluation process. If it is assumed that these drawings will not change during the evaluation process, the data set can be considered as quantitative, objective, and concrete. Additionally, this objective data set and calculations based on this data set do not reflect the intention of the jury members. As a result, the entropy method is thought to be more appropriate than AHP since the analysis will be based on only fixed quantitative data. 

Entropy, which is an objective method, also offers significant potential in enhancing the comprehensibility of subjective tendencies that involve uncertainty. The measurable properties of buildings can be calculated through the use of entropy, defined as factors, such as the following: shape, height, scale, color, silhouette, and materials used. Although previous studies have calculated the entropy of single or multiple factors of buildings, built environments, and abstract objects, it was observed that entropy values calculated based on factors had been evaluated independently [15,16,76]. The independent evaluation of entropy values of different factors contradicts opinions regarding the basic characteristics of entropy expressed in the literature. While, Medvedkov [44] argues that entropy becomes important in better understanding the complex relations, especially in cases where the number of relationships between factors is high. Klinger and Salingaros [77] emphasize that the fact that complexity is obtained by summing sub-components does not reflect the internal organization among components. Anderson [78] states that complex systems cannot be reduced to their components or the qualities of its’ components.

Evidently, municipal building projects are complicated design problems with many different inputs. For municipal building designs, many factors aside from aesthetic values, such as functionality, circulation, and size of spaces are all considered. In this context, overall entropy found by summing the entropy values of individual factors can only be applicable in cases where there is no relationship, ranking of significance, or weight among the factors. However, in design and jury evaluation phases of buildings, a number of factors establish relationships with each other to different degrees. Güzelci [79] investigated whether there is a relationship between calculated entropy values of individual factors in municipal building projects and the achievements they garnered in competitions; no consistent relationships between the individual entropy values and the success ranking of awarded projects were found.

Studies investigating the relationship between the levels of complexity and subjective responses in the literature indicate that the entropy values of buildings are directly related to the complexity people perceive, and their preferences [14,15,56,80,81]. On the other hand, researchers often work with the issue at hand by way of their own simple designs [14,15,16,76,82]. There is a lack of studies calculating the entropy values of complex buildings designed by other architects. In addition, there are few studies related to architectural programs and typologies while measuring entropy values of buildings [83,84].

The relationship between entropy value and subjective evaluation has been investigated in many studies and it has been emphasized that this relationship can be linear, asymptotic, or inverted U (Figure 1). Various researches have observed that the entropy values calculated vary in the range of 0–5.60 bits [14,16,82,85]. These entropy values can be considered low, and therefore produce asymptotic graphs [14,15,16]. The acceptance of the existence of an asymptotic graph between the entropy value and the subjective responses neglects the cases where entropy is above a certain value.

Based on the literature in the introduction; in this study, we developed the theoretical foundation of our method based on Berlyne’s [5] hypothesis that both low and high degrees of diversity are displeasing, while average degrees of diversity generate positive subjective responses. This hypothesis is supported by other scientific studies and it is recognized that this relationship results with an inverted U function. The calculation methods used in studies which measure the entropy of built environments through Shannon’s information entropy have been interpreted and used within this study [15,16,76] as applicable foundations of our method. Stamps [15,16,76] conducted subjective evaluations through using a survey and scaling method with respondents, however, our study conducted subjective evaluations through the competition jury. In the objective evaluation stage of the study, the entropy values are measured according to various factors. Although our entropy calculation methods are similar to Stamps’, the differences are the specialized factors to be measured, the combination of entropy values and jury evaluation results.

With the given shortcomings in mind, Entropy-Based Design Evaluation Model (EBDEM) was developed within this study to objectively analyze complex municipal building projects that participated in different competitions based on their multiple entropy values. To investigate high entropy values and inverted U relationships, the scope of this study examines complex buildings with a wide range of entropy values. This study does not create stimulants to examine complex buildings, rather, on the contrary, they are drawn through the drawings of existing projects.

Building on the findings by Güzelci [79], the single factors entropy values display no relationships to jury evaluation results, Weighted Overall Entropy (WOE) was introduced to consider such relationships between a wide range of different factors. WOE is obtained by multiplying entropy values of multiple factors by calculated weight coefficients; later the multiplications are summed for each building. EBDEM investigates the relationship between the WOE and the results of the jury assessment through an inverted U relationship. EBDEM uses a multi-objective genetic algorithm (MOGA) to calculate WOE values to each project and ranks them within an inverted U graph to produce outcomes similar to the jury assessment.

**Figure 2:**
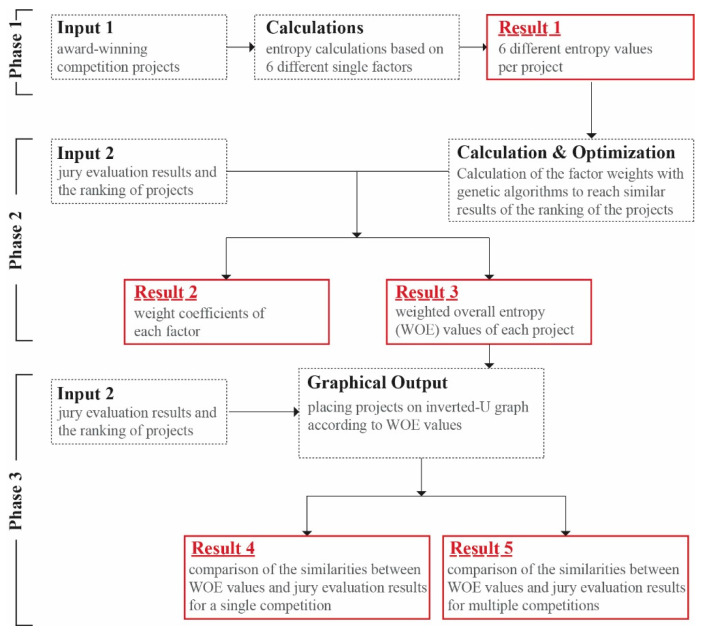
The general framework of Entropy-Based Design Evaluation Model (EBDEM).

The general framework of EBDEM is illustrated in Figure 2.

In the first phase of EBDEM’s framework (Figure 2), entropy values of the single factors are calculated by using methods described in Section 3.2. The input of Phase 1 is a representation of the projects as described in Section 3.1 (Input 1). Result 1 is the outcome of Phase 1; it provides the six different entropy values for each project.

In Phase 2, inputs are the jury evaluation results of the competitions and ranking of the projects (Input 2). At this stage, the relationship between Result 1 and the ranking of the projects was analyzed and as a result, the weight coefficients of each factor (Result 2) and the WOE value of each project (Result 3) were found.

In Phase 3, the input is identical to the input of the second phase (Input 2). The similarity of the results of the placing of the WOE values (as a result of the second stage) with the inverted U graph was examined; comparison of the similarities between WOE values and jury evaluation result were conducted for a single competition (Result 5) and for multiple competitions (Result 5).

### 3.1. Method Set-Up

This study aimed to measure the entropy of complex architectural typologies and in Section 3.1.1, the appropriate architectural typology was chosen among the complex typologies. To ensure accuracy in findings, the selection of competitions is important as this requires some logic, such as a select time period of competitions for appropriate comparison. Overall, for scientific accuracy, it is necessary to select appropriate samples rather than random samples. Afterward, competition projects with an award ranking were selected to be inputted into EBDEM. Within this study, the selected six factors to measure entropy were based on the juries most preferred criteria during the jury evaluation.

To measure and compare the entropy values of different buildings meaningfully, they should be represented within the same language. In Section 3.1.2, it was determined how the selected factors are represented with layers, shapes, and letters. Later, physical properties of buildings were interpreted on a computer by using Grasshopper.

Methods to measure the solid-void (factor 1), outline (factor 2), shape (factor 3), functional distribution (factor 4), and spatial flow entropy (factor 5) described in Section 3.2 were developed by re-interpreting the entropy measurement methods from previous studies. On the other hand, the concept of spatial relation entropy (factor 6) and the method of calculation was developed entirely within the scope of this study. Section 3.2 describes how to apply these six methods through a sample municipal building project. At the end of Section 3.2, the reMap operation is described, which enables the entropy values to be assigned to a specific range of values to be compared (Section 3.2.7).

In Section 3.3, the calculations are based on the entropy values of the single factors, which are calculated in Section 3.2. In Section 3.3, the WOE concept and the WOE calculation method, which are developed based on individual entropy values are described. While calculating the WOE values of buildings, multi-objective genetic algorithms constructed within the Galagapos optimization tool find weight coefficients according to the five given fitness functions.

#### 3.1.1. Selection of the Architectural Typology, Competition Projects, and Identification of Factors

Studies looking into the relationship between entropy and architecture, calculations are mostly performed on modular, repeating, distinct, and unambiguous components [17,76]. In this context, entropy can be calculated in buildings such as mass housing, schools, and municipal buildings which have an organized complexity. First, a preliminary study was conducted to select the building typology. For example, entropy measurements cannot be made because mass housing does not contain any functional diversity and schools have different architectural schemes and scale differences.

In this study, the reasons for the selection of municipal buildings for entropy calculation are listed below:

- consists of modular units with repeating shapes and dimensions so as to ensure functional requirements and to meet the size of the space identified;
- has a common functional diversity and organizational scheme;
- the use of predefined or well-known schemas to form the plan layout;
- constitutes a large number of resulted project archives which can be used to conduct entropy calculations.

In the scope of the study, we selected 42 municipal building projects for entropy measurements, which received various prizes in seven national architectural competitions organized as of 2015 in Turkey. The selected projects to which will be analyzed by EBDEM and the respective competitions they have participated in are as follows:

- Competition 1: Tekirdağ Metropolitan Municipality Building and Its’ Surroundings Architectural Project Competition (2015)—six projects;
- Competition 2: İzmir Konak Municipality Building and Its’ Surroundings Architectural Project Competition (2015)—six projects;
- Competition 3: Bornova Municipality Building and Its’ Surroundings Architectural Project Competition (2015)—six projects;
- Competition 4: Efeler Municipality Building Architectural Project Competition (2016)—six projects;
- Competition 5: İnegöl Municipality Building Architectural Project Competition (2016)—six projects;
- Competition 6: Van İpekyolu Municipality Building Architectural Project Competition (2016)—six projects
- Competition 7: Süleymanpaşa Municipality Building Architectural Project Competition (2017)—six projects.

There are many physical features (factors) in the projects which can be chosen to measure entropy. Since the relationship between the entropy values which are calculated on the basis of factors and the results of the jury evaluation, this study is of great importance to determine the factors affecting jury evaluation. The common features present in all municipal building projects and the criteria which were listed within the jury reports, the following factors to be measured for entropy in the study were identified: solid-void (factor 1), outline (factor 2), shape (factor 3), functional distribution (factor 4), spatial relation (factor 5), and spatial flow (factor 6).

#### 3.1.2. Representation of Factors

In previous studies, it is observed that visuals, drawings, or objects are re-coded to be input to entropy calculations [13,14,15,16,17,56,57]. Entropy calculations are highly related to how visualizations or drawings are done. To measure and compare the entropy values of different buildings meaningfully, they should be represented within the same language. The clear and consistent representation allows the buildings to be read visually and entropy calculations can be made without ambiguity. Furthermore, the level of detail of representations is an important parameter.

**Figure 3:**
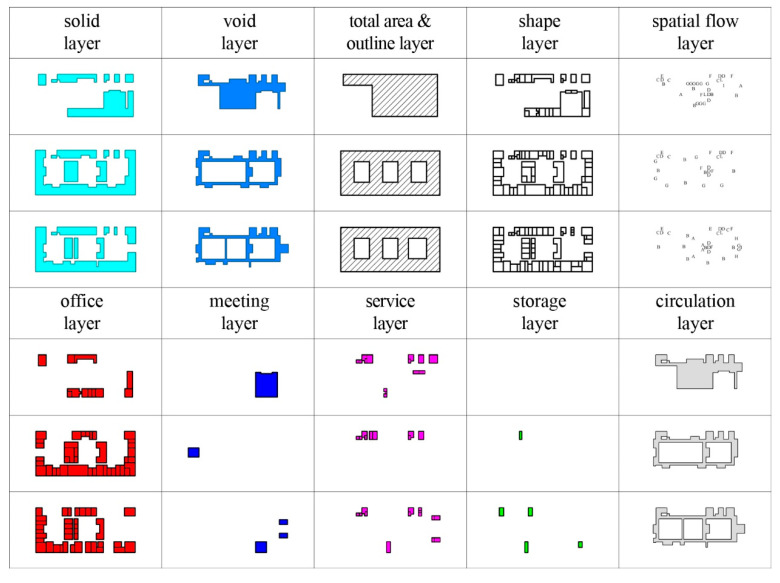
Representation of a variety of factors and its levels in different layers.

Floor plans in the project panels must be redrawn so as to include the layers referencing various physical features in order to calculate the entropy of six factors in the projects. Figure 3 illustrates the representations of the factors whose entropy will be calculated and the sub-levels of those factors as well as the names of the layers on which they are represented. Representation methods explained in Figure 3 can easily be distinguished from one another as they consist of shapes, colors, textures, and letters.

**Figure 4:**
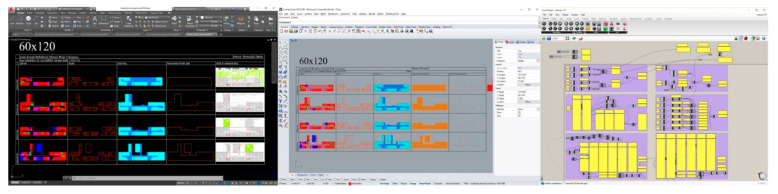
Transfer of project data through AutoCAD, Rhinoceros, and Grasshopper environments.

Raw data (floors plans) prepared as two-dimensional drawings on AutoCAD environment as per projects were transferred into Rhinoceros and were then converted processable on a computer environment through the use of the layer, shape, and letter recognition algorithms developed on Grasshopper visual scripting environment (VSE); and afterward, calculations were made (Figure 4).

An algorithmic system was developed to calculate entropy of six factors in 42 projects without user intervention to eliminate error and increase efficiency. Algorithms developed on the Grasshopper VSE possess the capability to recognize and use as inputs in entropy calculation all shapes, and letters drawn in different layers in two-dimensional drawing programmes. 

**Figure 5:**
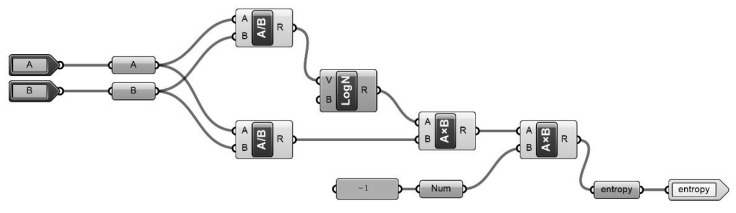
Definition of basic entropy equation in Grasshopper visual scripting environment (VSE).

As the first step of entropy calculations, the basic entropy equation formula was defined in Grasshopper VSE. Entropy calculation is conducted according to the probability of occurrence of an element (event) A, which can be defined as quantitative values in Grasshopper VSE, in the space of element (event) B (Figure 5).

### 3.2. Method 1: Entropy Calculation for a Single Factor

To calculate entropy of six factors in the municipal building projects, we reinterpreted the calculation methods used in previous studies, and when necessary, developed novel calculation methods. While solid-void and shape factors were used in previous research [16,17,76] to calculate entropy of three-dimensional abstract compositions, in this study to calculate entropy in two-dimensional floor plans and plan elements were used. While outline factor was used [16,57,80,85] to calculate the silhouette lines of buildings and cities, we reinterpreted it to be used for calculation of entropy of the outline of floor plans. Methods used to calculate the color entropy of two- and three-dimensional abstract compositions [14,15,76] were reinterpreted in this study to calculate the distribution of the functions represented by color. The method of using letters to represent the characteristics and transition between open spaces in site plans [12,16] was reinterpreted to calculate the entropy of interior spatial flow elements in municipal buildings which are represented by letters. Despite the lack of a method used to calculate spatial relation entropy in the literature, we developed a novel method to calculate entropy based on the ways in which distinct spaces in municipal buildings combine, based on Stiny’s [86] Froebel Blocks and Durhuus and Eilers’ [87] study into the spatial relation LEGOs form with one another.

In this section, individual entropy calculation methods based on solid-void, outline, shape, functional distribution, spatial relation, and spatial flow factors are explained in detail through the example of a municipal building project.

#### 3.2.1. Factor 1: Solid-Void Entropy

**Figure 6:**
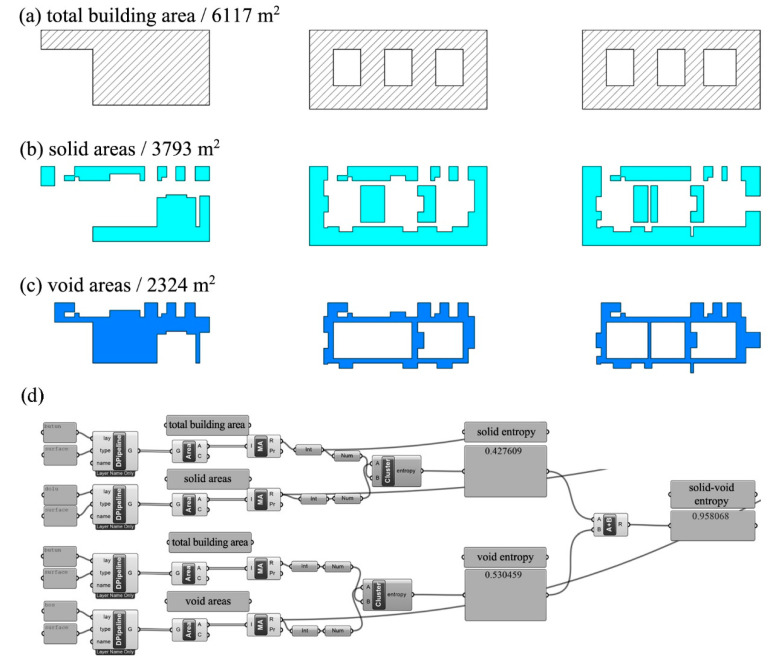
A sample calculation of solid-void entropy. (**a**) Total building area; (**b**) Solid areas; (**c)** Void areas; (**d**) Calculation of the solid-void entropy.

To calculate the solid-void entropy in a building, it is necessary to find the ratio of solid and void areas in all floor plans to the total building area. In the study, atriums, staircases, lifts, service shafts, and corridors were regarded as void areas, whereas all other spaces separated by doors were regarded as solid areas. The sample calculation illustrated in Figure 6 describes the total area of all the floors of the building calculated as 6117 m^2^, solid areas as 3793 m^2^, and void areas as 2324 m^2^ (Figure 6a–c). Having inserted the ratio of solid and void areas to the total building area in the entropy formula, we calculated the solid entropy as 0.427609 bits and the void entropy as 0.530459 bits. By summing these two values, we calculated the solid-void entropy of the building as 0.958068 bits (Figure 6d).

#### 3.2.2. Factor 2: Outline Entropy

**Figure 7:**
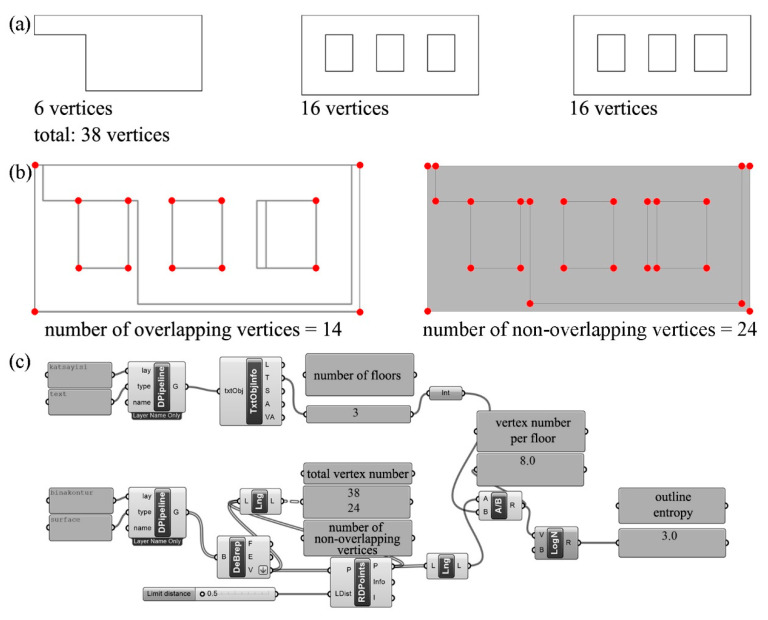
A sample calculation of outline entropy. (**a**) Number of vertices; (**b**) Number of overlapping and non-overlapping vertices; (**c)** Calculation of the outline entropy.

To calculate outline entropy, it is necessary to know the vertex number of the outer and, if any, inner contours forming the project’s floor plans. As shown in Figure 7, we added points to the vertices of the outer and inner outlines of the floor plans of the building and found the number of vertices in the floor plans to be six, 16, and 16, respectively (Figure 7a). Next, we superposed all floor plans and neglected 14 overlapping vertices. Subtracting the 14 overlapping vertices from the total number of vertices, we found the number of the non-overlapping vertices to be 24 (Figure 7b). The number of non-overlapping vertices divided by the total number of floors, three, produced the number of vertices per floor as eight. Finally, by calculating the logarithm in base two for the number of vertices per floor, we calculated the outline entropy of the building as 3 bits (Figure 7c).

#### 3.2.3. Factor 3: Shape Entropy

The municipal building projects investigated under the study, units such as manager rooms, secretary rooms, meeting rooms, service areas, and toilets were found to be repeated with unchanged size and form. In the scope of the study, the shape entropy calculation that Crompton [17] used for three-dimensional LEGO pieces was reinterpreted for municipal buildings composed of repetitive two-dimensional shapes.

To calculate shape entropy, it is necessary to identify how many different units exist in the floor plans and the quantity of each unit. In complex buildings consisting of many spaces, such as municipal buildings, it is difficult to count this manually. Therefore, a shape recognition algorithm was developed to count and distinguish different shapes from each other.

**Figure 8:**
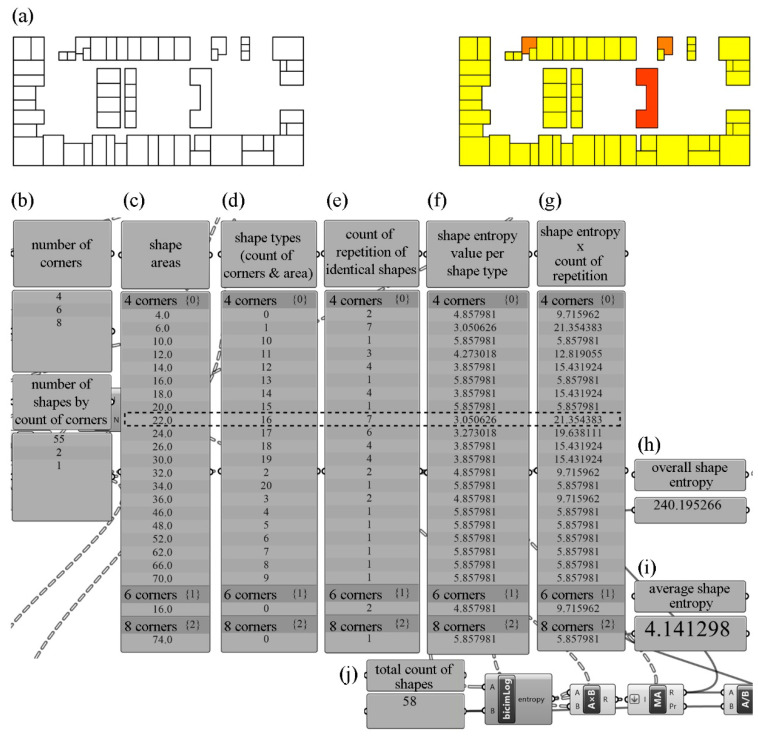
A sample calculation of shape entropy. (**a**) Colored shapes according to the corner numbers; (**b**) Number of corners and number of shapes by count of corners; (**c**) Shape areas; (**d**) Shape types; (**e**) Count of repetition of identical shapes; (**f**) Shape entropy value per shape type; (**g**) Multiplication of shape entropy and count of repetition; (**h**) Overall shape entropy; (**i**) Average shape entropy; (**j**) Total count of shapes.

In the shape entropy calculation based on a sample floor plan in Figure 8, first, the number of corners of all closed shapes were counted, and the corner numbers of shapes were found according to the number of vertices in each shape (Figure 8a). Once the shapes were grouped according to their corner numbers, it was calculated that the floor plans included shapes with four, six, and eight corners and that there were 55, two, and one of these shapes, respectively (Figure 8b). The shapes grouped according to their corner numbers were also grouped by the length of their perimeter and their areas. Later, the repetition count of the types of grouped shapes and the reoccurrence of identical shapes were noted. For example, it was calculated that there were seven identical “no.16”-type shapes, having four corners and an area of 22 m^2^ (Figure 8c–e). When an entropy calculation was performed according to the incidence of shape “no. 16”, which repeated seven times in the floor plans, on the total space of shapes, composed of 58 shapes, it was found that shape “no. 16” had a value of 3.050626 bits per part (Figure 8f). As seven of the particular shape with an entropy value of 3.050626 bits, they, in total, corresponded to entropy of 21.354383 for the building (Figure 8g). Repeating the same procedures for all shapes, the overall shape entropy of the building was calculated as 240.195266 bits (Figure 8h). Finally, dividing the overall shape entropy value of 240.195266 by the total count of shapes, 58, produced the average shape entropy value for the building, which was 4.141298 (Figure 8i,j).

#### 3.2.4. Factor 4: Functional Distribution Entropy

To calculate the functional distribution entropy value and compare the entropies of buildings using a standard method, buildings must have an equal number of functions and the same functions. Upon reviewing 42 municipal building projects under the study, five main functions were identified: offices, meeting rooms, archives, service areas, and circulation areas.

**Figure 9:**
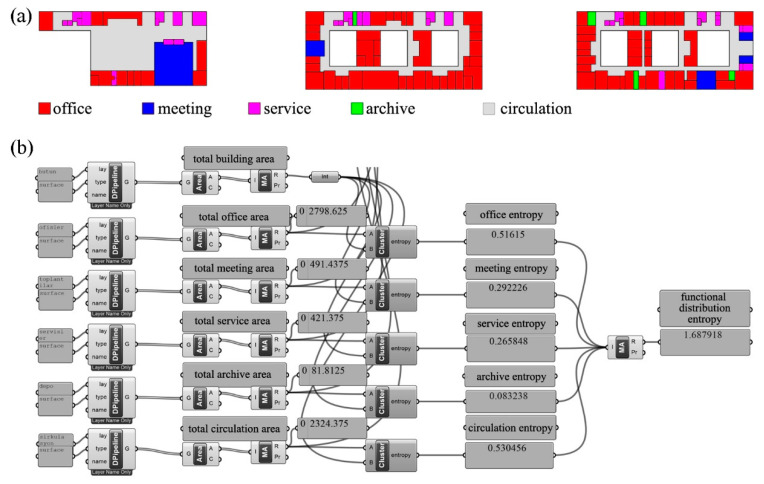
A sample calculation of functional distribution entropy. (**a**) Representation of the functions with colors; (**b**); Calculation of the functional distribution entropy.

In the sample calculation given in Figure 9a, after the functions were represented by different colors, the areas of spaces where each function was observed were summed as 2798.625, 491.4375, 421.375, 81.8125, and 2324.375 m^2^, respectively. The entropy value for each function was calculated as 0.516150, 0.292226, 0.265848, 0.083238, and 0.530456 bits, on the basis of the ratio of the sum of the area of all the units in a given function to the total area of the building. By summing the calculated entropy values for five functions, the functional distribution entropy of the total building was calculated as 1.687918 bits (Figure 9b).

#### 3.2.5. Factor 5: Spatial Relation Entropy

**Figure 10:**
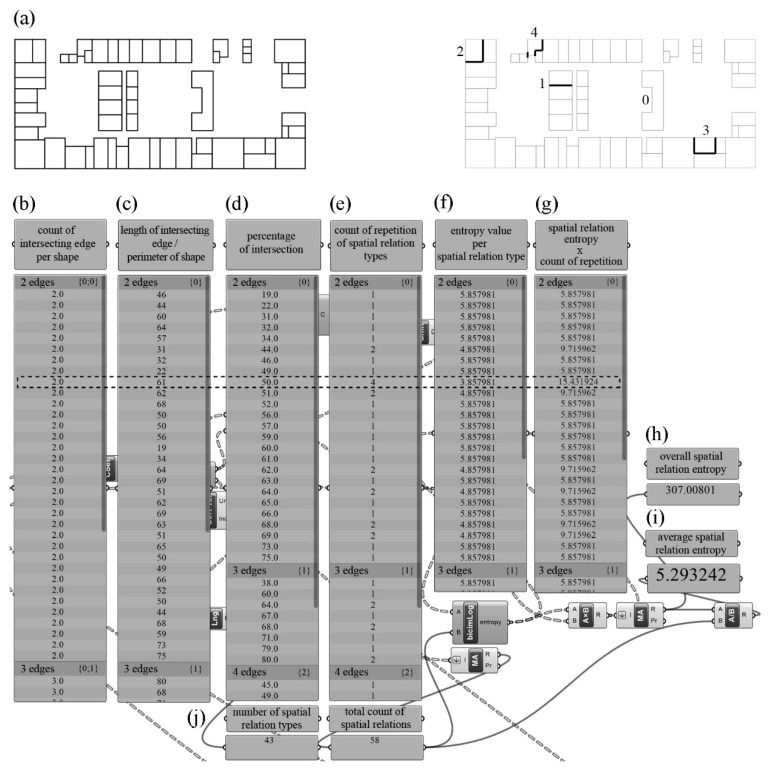
A sample calculation of spatial relation entropy. (**a**) Shapes and number of intersecting edges; (**b**) Count of intersecting edge per shape; (**c**) Ratio of the length of the intersecting edge to the perimeter of shape; (**d**) Percentage of intersection; (**e**) Count of repetition of spatial relation types; (**f**) Entropy value per spatial relation type; (**g**) Multiplication of spatial relation entropy and count of repetition; (**h**) Overall spatial relation entropy; (**i**) Average spatial relation entropy; (**j**) Number of spatial relation types and total count of spatial relations.

Through the review of floor plans of the municipal building projects, it was observed that two-dimensional closed shapes create a variety of diverse spatial relations with one another. To calculate spatial relation entropy, the spatial relations that each shape forms with all the other shapes were listed and the repetition number of the types of relations were ascertained. Therefore, an algorithm was developed to calculate spatial relation entropy on the basis of types of the intersection and the repetition count of intersection types for all shapes (Figure 10).

To calculate the spatial relation entropy through the floor plans, first, one needs to identify how many edges of the units intersect with other units. The sample floor plan in Figure 10a expresses the number of intersecting edges. According to the calculation performed by the algorithm, it was found that some shapes formed spatial relations with other units on edges 1, 2, 3, and 4; and these shapes were grouped in line with the count of intersecting edges per shape (Figure 10b). The next step calculated the ratio of the length of the edges of each unit that intersects with other units to the perimeter of the shape (Figure 10c). Based on the number of intersecting edges and the percentage of the intersection, the number of spatial relation types on the floor plan was calculated as 43 (Figure 10d,j). Figure 10e lists the count of repetition of spatial relation types on the floor plan. The highlighted row in Figure 10 describes a shape that intersects with other units on two edges. The ratio of the total length of the intersecting edges of the selected shape to the perimeter of the shape was calculated as 50%. It is observed that four of the 58 spatial relations which the shapes on the floor plan form are both formed on two edges and have a ratio of 50% (Figure 10d,e). As a result of the logarithm calculation in base 2, the entropy value of the spatial relation that is repeated four times on a relation space of 58 relations was calculated as 3.857981 bits (Figure 10f). By multiplying the entropy value of 3.857981 per selected spatial relation by the count of repetition of four, the entropy produced by the spatial relation type was calculated as 15.431924 bits (Figure 10g). By summing the results of the multiplications for each spatial relation type, the overall spatial relation entropy was calculated as 307.008010 bits (Figure 10h). Finally, by dividing the overall spatial relation entropy value by the total count of relations, 58, the average spatial relation entropy of the floor plan was calculated as 5.293242 bits (Figure 10i,j).

#### 3.2.6. Factor 6: Spatial Flow Entropy

Apart from the closed spaces for which shape entropy was calculated, the municipal buildings reviewed contained spatial flow elements such as hallways, general corridors, service corridors, lifts, fire stairs, stairs, inner corridors, foyers/waiting areas, stairway landings, bays, which related to each other physically and visually. As these units could vary in shape and size, they were represented by letters rather than shapes. Similar to the approach Stamps [16] used for site plans, letter sequences represented the spatial flows on the floors.

A common language was developed to represent the spatial flow elements in this study. The entrances and exits were represented by the letter “A”, general corridors by “B”, service corridors by “C”, lifts by “D”, fire stairs by “E”, stairs by “F”, waiting areas by “G”, inner corridors by “H”, hallways by “I”, foyers by “J”, bays by “K”, and stairway landings by “L”.

**Figure 11:**
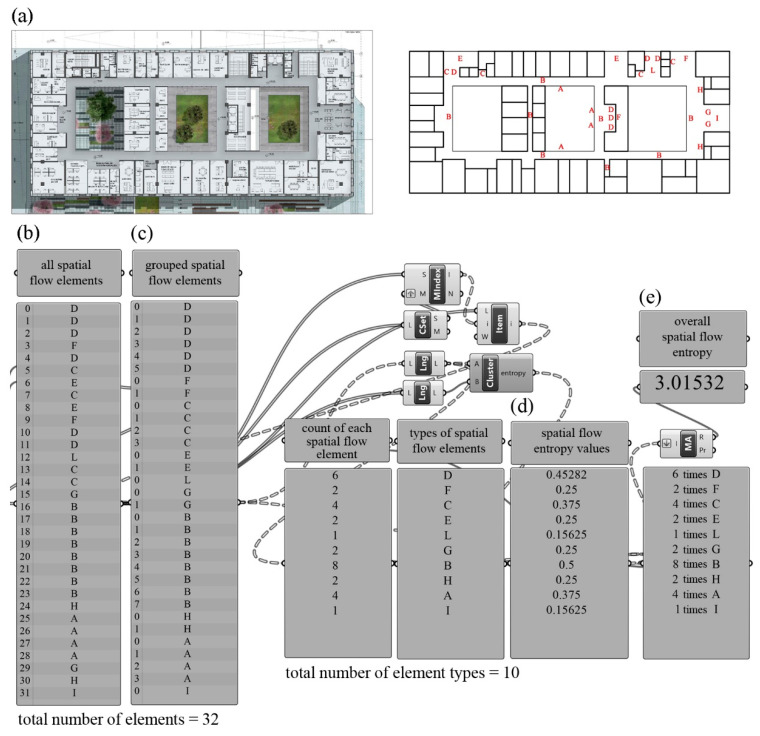
A sample calculation of spatial flow entropy. (**a**) Floor plan and spatial flow elements; (**b**) All spatial flow elements; (**c**) Grouped spatial flow elements; (**d**) Spatial flow entropy values; (**e**) Overall spatial flow entropy.

In the sample calculation in Figure 11, first, the spatial flow elements were placed on the floor plan (Figure 11a). Then, all spatial flow elements were converted into a string made of 32 letters (Figure 11b). The elements in the sequence were grouped according to the letters and the numeric distribution of each different spatial flow elements encountered in the project was ascertained (Figure 11c). By inserting the ratio of the repetition numbers of all elements to the total number of elements into the basic entropy equation, spatial flow entropy values were calculated for all elements individually (Figure 11d). Finally, by summing the entropy values calculated for all of each element, the overall spatial flow entropy of the floor plan was calculated as 3.01532 bits (Figure 11e).

#### 3.2.7. Remapping the Single-Factor Entropy Values

**Figure 12:**
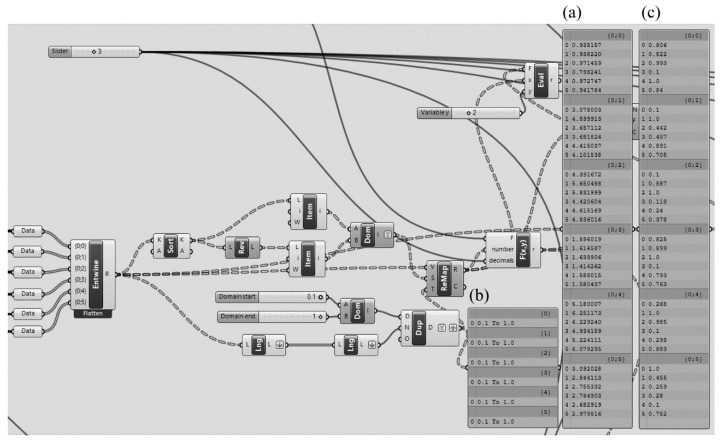
The structure of reMAP algorithm developed in Grasshopper VSE. (**a**) List of calculated entropy values; (**b**) Value range for remap operation; (**c**) Remapped entropy values.

The entropy values calculated on the basis of six different factors in the projects differ from one another quite widely in terms of numeric size. For example, while the solid-void entropy value varied in the range of 0–1 bit, the functional distribution entropy was in the range of 0–2.32 bits. To prevent meaningless results to be produced following the multiplication of the entropy values by weights and then summing the outcomes, the entropy values for all factors were adjusted and remapped into a common value range. The stages of the reMap operation are represented in Figure 12. The algorithm firstly lists all individual entropy values calculated (Figure 12a). The value range into which values would be remapped and assigned was determined as 0.1–1 (Figure 12b). Then, all the single-factor entropy values calculated were reassigned to the range of 0.1–1, with the largest value remapped to 1 and the smallest to 0.1 (Figure 12c).

### 3.3. Method 2: Entropy Calculation for Multiple Factors

**Figure 13:**
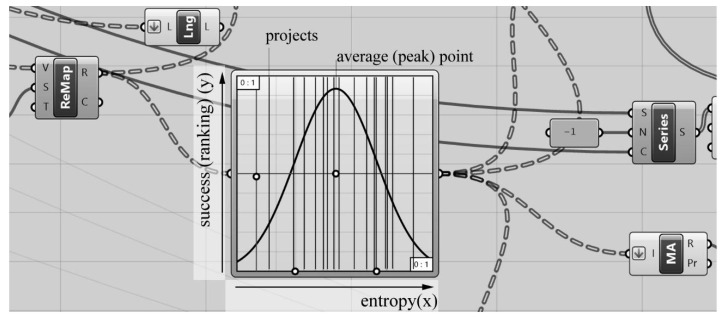
Representation of projects on inverted U graph in Grasshopper environment.

We discussed the effects of the entropy value level to subjective evaluation in Section 2.1. In this context, it is observed that an average entropy value leads to positive feedback but when the entropy value deviates from the average in a positive or negative direction along the X-axis, the positive feedback level such as preference decreases, and an inverted U graph is occurred (Figure 1 and Figure 13).

Single factor entropy values of competition projects can be easily obtained by applying the methods explained in Section 3.2. However, it is not possible to evaluate buildings through overall entropy values obtained by directly summing these individual factor entropy values without considering the relationship between them. Therefore, we developed a new concept (WOE) that considers the relationships of factor weights in this study.

#### 3.3.1. Weighted Overall Entropy (WOE)

Entropy values obtained by multiplying the single factor-entropy values of a building by different weights and by summing the values then is defined as the Weighted Overall Entropy (WOE). In this study, factor weight coefficients describe the importance degree of the criterion used in jury evaluation. Displayed in Table 1, by multiplying the entropy values of all factors (a, b, c, d, e, f) of municipal building projects by constant coefficients (w1, w2, w3, w4, w5, w6) and then summing them, a building’s WOE is calculated.

#### 3.3.2. Fitness Functions and Galapagos as an Optimization Tool

In this study, we aimed to place the projects into the inverted U graph according to their WOE values. The fitness functions to be used in WOE calculations, based on the hypothesis that an average level of entropy creates positive responses, are listed as follows:

- Fitness Function 1: The first-prized project is positioned closer to the peak of the inverted U graph than all the other projects.
- Fitness Function 2: The second-prized project is positioned closer to the peak of the inverted U graph than the other projects except for the first-prized project.
- Fitness Function 3: The third-prized project is positioned closer to the peak of the inverted U graph than the other projects except for the first-prized and the second-prized project.
- Fitness Function 4: The honorable mention projects are positioned on the inverted U graph farther away from the first-prized, second-prized, and third-prized projects.
- Fitness Function 5: The honorable mention projects are positioned on the inverted U graph without a ranking among them (Figure 13).

**Figure 14:**
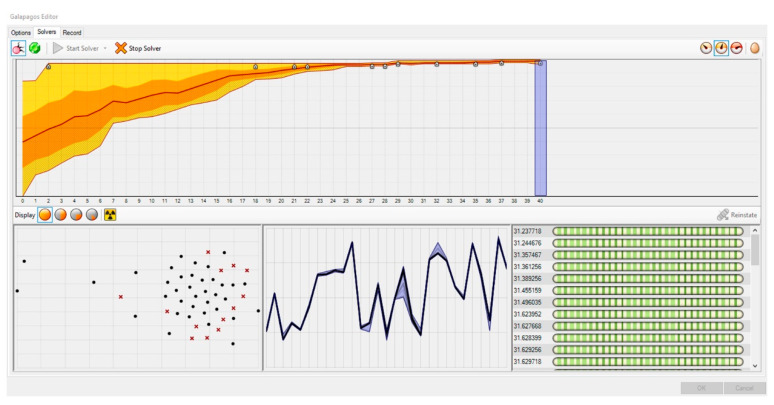
Interface of Galapagos solver during the optimization process.

In order to place the projects on the inverted U graph, the calculations have to be done according to multiple objectives (fitness functions) simultaneously. Problem-solving tools based on genetic algorithms reflect the evolutionary principles observed in nature and search for results by running several operations in parallel. Problem solver optimization tools embedded in VSE are used for these operations; the Grasshopper VSE possesses such optimization tools as Galapagos, Goat, and Octopus within it [88]. Although Galapagos, developed by David Rutten, performs single-objective evolutionary optimizations by using genetic algorithms, it can also solve multi-objective optimization problems if a number of different objectives are defined as a single fitness function [89,90,91]. Due to the flexibility of Galapagos; it was used to combine the five fitness functions and to optimize the weight coefficients (Figure 14).

The multi-objective non-dominant sorting algorithm, developed under the study, multiplies six individual entropy values calculated for each project by six weight coefficients. While the project’s entropy values calculated on the basis of factors remain constant, weight coefficients are continuously changed by the genetic algorithm to conduct the multiplications. By changing the weight values constantly, multi-objective genetic algorithm (MOGA) makes searches to position the WOE value to the intended points on the inverted U graph, in other words, to the position in line with the results of the jury evaluation.

In the classical genetic algorithm approach, the determination of the weights of fitness functions affect the solution sets to a great extent and guide the results. In this study, however, competition results, taken as solution sets, and the method developed set the genetic algorithm free rather than guide it. This allows an analysis of the competition evaluation process by calculating the weights of the factors and the WOE values based on the factors.

## 4. Investigation of the Relationship between Objective and Subjective Measurements through EBDEM: A Case Study on Municipal Buildings

In the first stage of EBDEM, entropy values were calculated for six factors in all projects through the use of the methods described in Section 3.2. Drawings representing different physical features of the buildings were transferred from the AutoCAD environment into Rhinoceros. Then, data on Rhinoceros were interpreted on the Grasshopper VSE and six entropy values were calculated for each of the 42 projects. It is observed that there is no numeric similarity in terms of a range between the factor 1, 2, 3, 4, 5, 6 entropy values of the projects reviewed under the study. Then, the calculated entropy values are remapped to new values in the range of 0.1–1 (Table 2).

**Figure 15:**
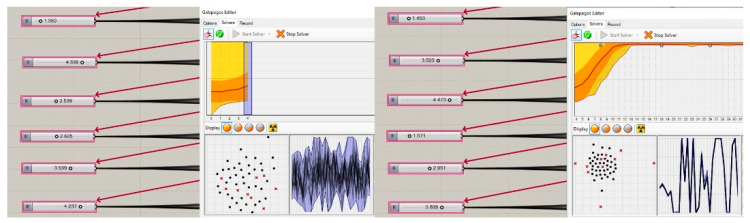
The search and fix process of the weight coefficients in Galapagos.

Afterward, five fitness functions were incorporated into a single fitness function on Galapagos. The fitness function defined for optimization based on multiple factors in the study aims to first multiply these remapped values by weight coefficients and hence calculate the WOE and assigns the projects to the assumed points within the inverted U graph. After MOGA conducts countless multiplications of the projects’ entropy values to meet the fitness function, the weight coefficients were fixed, with the WOE values synced with the closest possible jury results (Figure 15).

**Figure 16:**
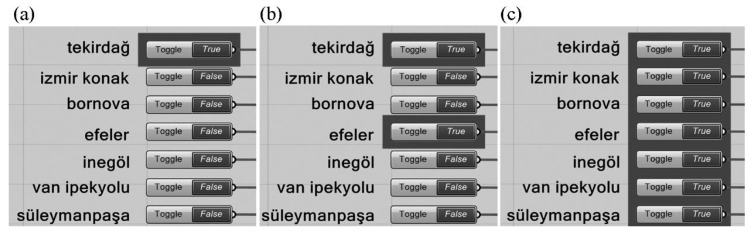
The module used to select competitions to be included in the entropy calculation. (**a**) Calculation for Tekirdağ competition; (**b**) Calculation for Tekirdağ and Efeler competitions; (**c**) Calculation for all competitions.

EBDEM has the ability to perform weight coefficient and WOE calculations for any given number of competitions. A module consisting of seven buttons was developed on Grasshopper VSE to identify the competitions to be included in the calculation. The buttons used in the module have a set of two values, either “true” or “false”—“true” signifying that a competition has been included in the calculation and “false” if excluded. For example, while the awarded projects in the Tekirdağ competition were included in the calculation as shown in Figure 16a, a total of 12 awarded projects in Tekirdağ and Efeler competitions were included in the calculation in Figure 16b. In Figure 16c, however, as all the buttons had the value “true”, all the awarded projects in all competitions were included in the calculation.

Two different experiments were conducted with EBDEM in the study. First, EBDEM was used to rank six projects awarded in a single competition. By finding the appropriate weight coefficients for six factors in six projects, MOGA aimed to render WOE values similar to the jury evaluation results. This method was repeated for seven competitions; six weight values and WOE values, for each competition, were calculated. Second, 42 projects awarded in seven competitions were simultaneously optimized by MOGA. At this stage, the individual entropy values of all projects were multiplied by the weight values, with a goal to ensure that the WOE value reflected in the inverted U graph will bear similar results to jury evaluation outcomes. Using this method, six weight values in total for all competitions were calculated.

### 4.1. Experiment 1: Weight Coefficient and WOE Calculation for a Single Competition

**Figure 17:**
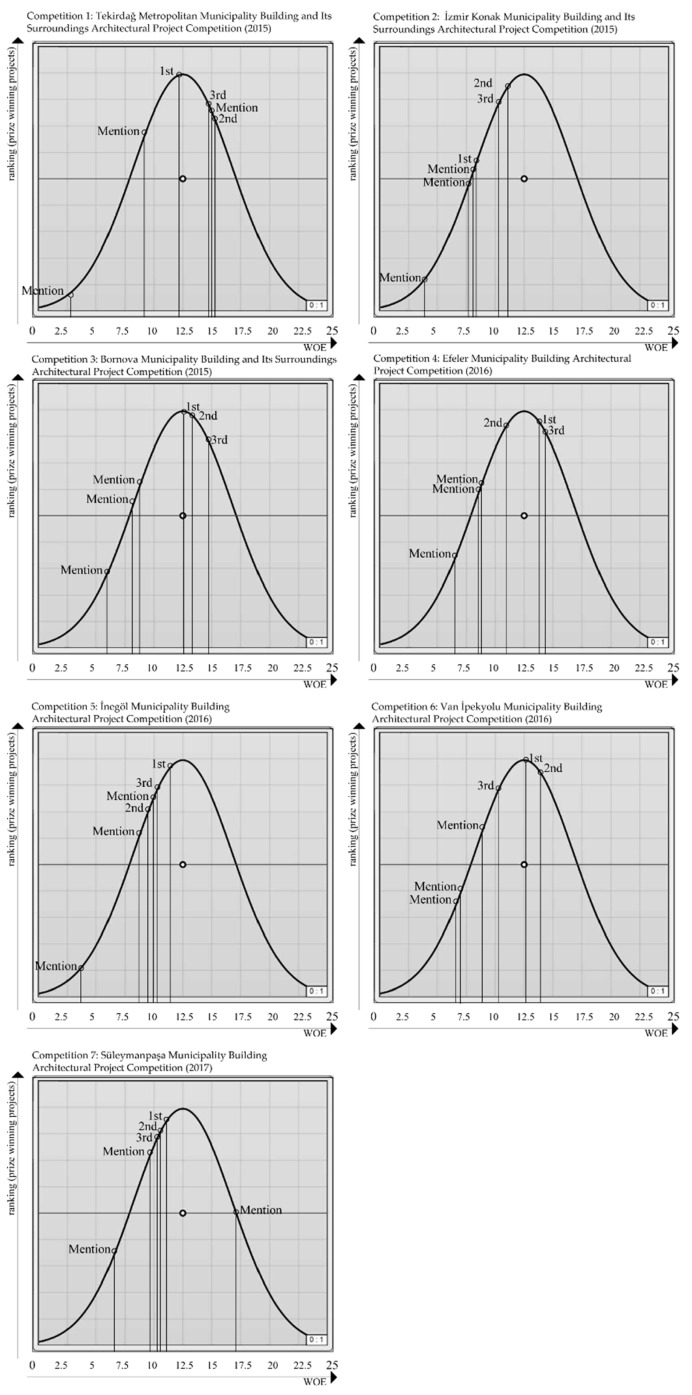
Illustration of WOE values on inverted U graphs for each single competition.

While calculating WOE of multiple factors, we first aimed to obtain the ranking specified in the fitness function by assigning weights to factors in six projects awarded in each competition so that these projects will first be ranked among themselves. By means of the buttons indicated in Figure 16, each competition was optimized independently from one another and the weight coefficients for each factor were calculated. The breakdown of the six weight values calculated for each competition following Galapagos optimization is presented in Table 3. The points that the projects hold on the inverted U graph in line with the WOE values obtained by multiplying the weight coefficients by the entropy values of factors and then summing the results are presented in Figure 17.

### 4.2. Experiment 2: Weight Coefficient and WOE Calculation for Multiple Competitions

Investigating multiple award-winning competition projects by using the same method may result in significant findings of that specific architectural typology and programme. Although both experiments followed the exact method to calculate weight coefficient and WOE values; Experiment 2 encompassed a greater sample size with an objective to achieve comprehensive findings.

**Figure 18:**
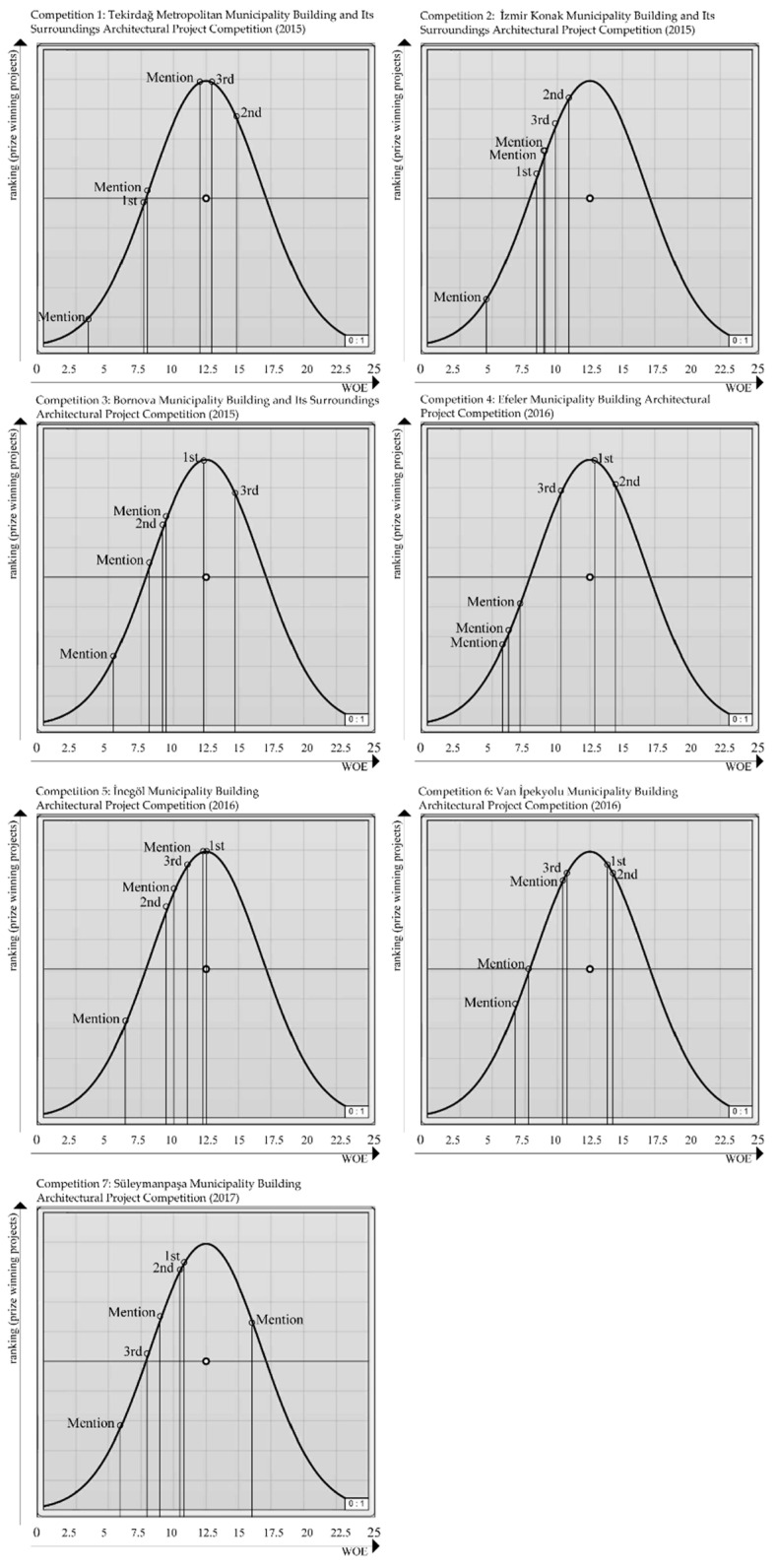
Illustration of WOE values on inverted U graphs for multiple competitions.

While calculating the weight coefficients for seven competitions, 42 projects were simultaneously optimized. Then, six weight coefficients were simultaneously calculated for all the projects so as to meet the fitness functions. Galapagos calculated the weight values as 1.453, 3.503, 4.473, 1.571, 2.591, 3.805, respectively, for each of the factors (Table 4). Using the method specified in Table 1, the WOE values were calculated for each project. The points that the WOE values indicated in Table 4 hold on the inverted U graph are provided in Figure 18.

### 4.3. Results of the Experiments

As a result of Experiment 1, the findings state a 100% similarity was obtained among the projects placed on the inverted U graph in line with the jury evaluation results of the following competitions, according to WOE values: Competition 3, Competition 4, Competition 6, and Competition 7. The ranking of other projects on the inverted U graph in line with WOE values was as follows: first-prized, third-prized, mention, second-prized, mention, mention for Competition 1; second-prized, third-prized, first-prized, mention, mention, mention for Competition 2; and first-prized, third-prized, mention, second-prized, mention, mention for Competition 5 (Figure 17). According to the results in Table 3, factor weight values for each competition differ so as to meet the fitness function. As a result, we achieved a total of 42 weight coefficients for all projects and six for each project.

As a result of Experiment 2, the findings state that Competition 6 was the only competition with 100% similarity with jury evaluation results. The remaining competitions analyzed were similar to jury evaluation results to some degree. The ranking on the inverted U graph in line with WOE values was as follows: third-prized, mention, second-prized, mention, first-prized, mention for Competition 1; second-prized, third-prized, mention, mention, first-prized, mention for Competition 2; first-prized, third-prized, mention, second-prized, mention, mention for Competition 3; first-prized, third-prized, second-prized, mention, mention, mention for Competition 4; first-prized, mention, third-prized, mention, second-prized, mention for Competition 5; first-prized, second-prized, mention, mention, third-prized, mention for Competition 7 (Figure 18). According to the results in Table 4, factor weight values for all competitions are the same, so as to meet the fitness function. As a result, we achieved a total of six weight coefficients for all projects.

While Table 3 and Table 4 display the weights calculated by using MOGA and the WOE values calculated on the basis of the weights, Figure 17 and Figure 18 present the positioning of the projects on the inverted U graph in line with their WOE values. It was observed that the positioning on the inverted U graph of the projects based on the WOE values obtained by analyzing a single competition bears more successful results than when multiple competitions are calculated simultaneously.

In Experiments 1 and 2, MOGA was inconsistent in approaching all the WOE values of the first-prized projects to the identified average value of 12.5 bits. It was observed that MOGA searches the weight values and WOE values not only for first-prized projects but also for the other awarded projects simultaneously. In both experiments, rather than obtaining perfect results for a single project, MOGA searches for optimum results for all the projects and ranks them according to five given fitness functions.

The projects awarded in competitions do not have to be within a certain WOE value range. For example, while the WOE values of the six projects awarded in one competition might vary between nine and 13 bits or three and 15 bits and despite a difference of four or 12 bits, the rankings may be appropriate. The lack of a range of WOE values which might be indicated to be appropriate makes it impossible to make any percental interpretation of the similarity between the WOE values obtained through calculations and the results of the competition.

## 5. Discussion

The major goal of this study was to investigate the relationship between the multiple entropy values obtained by the objective measurement of competition projects and success achieved as a result of the subjective evaluation of an architectural competition jury with the proposed model, EBDEM. As emphasized in the introduction, the two principal conclusions and expected results of this study may be summarized as follows.

Factors, competitions, and the number of projects were the constraints of this study. It is impossible to come to definitive conclusions about the subjective foundations of jury evaluation through examination of six factors. The selected factors examined within the case study are the common evaluation criteria obtained from jury reports within the selected architectural competitions. However, evaluation criteria are not limited to solely these identified factors. Based on the findings of the study, it is estimated that an increase in the number of factors and competitions may increase the predictability of jury evaluation results and the award-winning potential of the projects.

Within architectural competitions, objective and subjective evaluations are done simultaneously. Although entropy is used to make objective measurements, numerous studies associated entropy with subjective assessments. Within this study, relationships between subjective evaluation of jury members and objective entropy calculation were analyzed. In calculations based on many factors, we investigated which factors are important and have a greater impact on evaluations. These measurable factors do not impact the evaluation independently; rather, they relate with other unmeasurable qualities and have an impact collectively. EBDEM can analyze the level of objectivity of jury evaluations on the basis of the entropy values calculated based on measurable qualities of competition projects, only if the jury evaluations are based on projects’ entropy values. The justification for EBDEM’s decrease in accuracy when applied to many competitions at the same time is the increase in evaluation criteria of different competition juries. Changes in sample group or competitions lead to a change in observed single-factor entropy values, weight coefficients, and WOE values. As a consequence, subjective means of evaluation, like jury assessment, can be associated with an objective and computable evaluation model like entropy, to some degree.

EBDEM was applied to a total of 42 municipal building projects awarded in seven different competitions. Using the single factor entropy calculation methods, which constitute the first module of EBDEM, entropy values of six factors of 42 projects were calculated. The results indicated a numeric difference in range between projects’ entropy values. Furthermore, the overall entropy values, obtained by summing the single factor entropy values of the projects, do not demonstrate any numeric similarity. Therefore, it was concluded that there was no single or overall entropy value which could be considered optimum for municipal buildings.

In this study, we suggested that examining a large number of award-winning projects in different competitions within the same framework may be useful in obtaining important findings of selection criteria. EBDEM found which factors are influential to which degree, with an assumption that the jury makes an evaluation according to entropy values with varying weights.

Another important finding of this study is that complex municipal buildings with high entropy values support the inverted U graph between entropy values and jury evaluation results. If the inverted U graph relationship proves to be true, then further design alternatives can be modified to meet the average entropy values given in the inverted U graph.

Under this study, EBDEM evaluated finalized architectural projects which cannot be altered post-submission. The design stage, however, is one that is open-ended and dynamic, allowing projects to change and evolve over time. Hence, by adding new features to EBDEM, one can develop MOGA-based expert systems that can produce designs by bringing the entropy values to the levels specified by the designer in line with the defined fitness functions. Therefore, EBDEM can be used not only to analyze existing buildings but also to help new building designs evolve.

It is not possible to analyze all factors which relate to human preferences in a single study. Individual studies in the literature have often focused on several factors and as a consequence, researchers have discovered findings of relationships between a large number of factors and subjective responses such as arousal, pleasure, and preference. Under this study, EBDEM evaluated finalized architectural projects which cannot be altered. The design stage, however, is one that is open-ended and dynamic, allowing projects to change and evolve. Hence, by adding new features to EBDEM, one can develop MOGA-based expert systems that can produce designs by bringing the entropy values to the levels specified by the designer.

The experiments made, and the model developed in the study differs from previous studies in the literature because of its features listed below:

- Entropy calculation of complex architectural typologies designed by other architects.
- Calculation of factor weights taking into consideration the relations between multiple entropy values.
- Analysis of the relation between projects with a wide WOE range and the success they garnered in competitions.

In many genetic algorithm applications, first, a great number of alternatives are created. Then the designer selects among the designs on the basis of a variable number of fitness functions defined. In this study, however, genetic algorithms were used not to create design alternatives but to rank the projects whose digitizable features were analyzed with the entropy method based on many multiple fitness functions. MOGA was used to optimize the projects in the inverted U graph similar to the jury evaluation results according to the WOE values, which were outcomes of EBDEM. MOGA aims to find the assumed WOE values by multiplying the entropy values of different factors by variable weight coefficients.

Considering that the factors related to each other during the evaluation stage, we used MOGA to find the weight coefficients of the factors and WOE values of the projects with an objective of achieving results similar to those of the jury evaluation. The points observed on the inverted U graph based on their calculated WOE values for each project were compared with competition results. Accordingly, it was found that single-competition weight and WOE optimizations were more similar to jury evaluation results than a multiple-competition optimization.

There are some potential limitations to consider in this study. The objective of the fitness function was to obtain optimum results suitable for all fitness functions rather than perfect results. To obtain optimum solutions, there must not be any inconsistencies between fitness functions. Within the preliminary stages of this study, all projects, whether awarded or not, were included. Since MOGA brought the weight coefficients close to zero while decreasing the rank of non-awarded projects, ultimately, the non-awarded projects were excluded. In this case, because two conflicting fitness functions were defined for awarded and non-awarded projects, the algorithm could not function effectively.

Furthermore, EBDEM has the capability to conduct entropy calculations without the need to depend on scale, size (square meters), number of floors, units forming the building, or project numbers. In this context, EBDEM, developed via the plans of municipal buildings, can be applied to any type of building. It is possible to use EBDEM for entropy calculation of buildings of a period, by a specific architect, or of similar quality. EBDEM can be applied to building typologies with a complex organization scheme such as hospitals, airports, cultural centers, or schools, or to larger-scale neighborhoods and towns.

### 5.1. Further Improvements

Representation plays an important role in calculations for buildings and their components in design disciplines. In the study, different aspects of buildings were represented on different layers, using lines, planes, and letters and were hence distinguished from one another. Therefore, it would be possible to calculate entropy by means of the physical features defined in the Building Information Model (BIM), the data from which are stored in different layers, without the need for any drawing or parsing into different layers. Algorithms devised, and calculations made on the Grasshopper VSE, interoperable with Rhinoceros, in the study can also be performed on Revit and Dynamo environments working on the basis of the BIM.

Clark and Pause [92] state in the foreword to “Precedents in Architecture” that beyond learning architecture and understanding elements, analyzing buildings also contribute to the design stage. Besides conducting objective analyses, the entropy calculation methods developed in the study provide the opportunity to read the buildings on different layers and to obtain important data by drawing meaningful comparisons similar to shape grammar, fractal analysis, and spatial syntax methods. It is thought that grasping the solutions and alternatives that similarities introduced into municipal buildings might also inform the design stage.

In further exercises, we aim to develop EBDEM into an expert system that produces design alternatives and simultaneously tests the designs produced. In this study, multiple detailed entropy calculations were performed by algorithms. It is expected that the expert system will also be equipped with artificial intelligence and in addition to making calculations, will also solve design problems with the help of “knowledge” present in big-data systems.

## Figures and Tables

**Table 1:** Proposed calculation method and formula of Weighted Overall Entropy.

Competition	Award/Ranking	Factor 1 Weight Value (w1)	Factor 1 Entropy Value (a)	Factor 2 Weight Value (w2)	Factor 2 Entropy Value (b)	Factor 1 Weight Value (w1)	Factor 3 Entropy Value (c)	Factor 1 Weight Value (w1)	Factor 4 Entropy Value (d)	Factor 1 Weight Value (w1)	Factor 5 Entropy Value (e)	Factor 1 Weight Value (w1)	Factor 6 Entropy Value (f)	Weighted Overall Entropy (WOE)
competition 1,…, n	1st, 2nd, 3rd,…, mention	w1	a1, a2, a3,…, an	w2	b1, b2, b3,…, bn	w3	c1, c2, c3,…, cn	w4	d1, d2, d3,…, dn	w5	e1, e2, e3,…, en	w6	f1, f2, f3,…, fn	WOE1, WOE2,…, WOEn
Weighted Overall Entropy = (w1 × an) + (w2 × bn) + (w3 × cn) + (w4 × dn) + (w5 × en) + (w6 × fn) = WOEn														

**Table 2:** Calculated entropy values of six factors and their corresponding remapped values ranging from 0.1 to 1.

Competition	Status	Factor 1 Entropy	Factor 1 reMAP	Factor 2 Entropy	Factor 2 reMAP	Factor 3 Entropy	Factor 3 reMAP	Factor 4 Entropy	Factor 4 reMAP	Factor 5 Entropy	Factor 5 reMAP	Factor 6 Entropy	Factor 6 reMAP
Tekirdağ Competition	1st	0.935157	0.806	3.078003	0.1	4.391672	0.1	1.596019	0.825	5.180007	0.268	3.092028	1
	2nd	0.93822	0.822	4.599913	1	5.650488	0.887	1.614587	0.899	6.251173	1	2.844113	0.455
	3rd	0.971459	0.993	3.657112	0.442	5.831999	1	1.639906	1	6.22924	0.985	2.755332	0.25
	men.	0.798241	0.1	3.681824	0.457	4.420604	0.118	1.414262	0.1	4.934159	0.1	2.764903	0.28
	men.	0.972747	1	4.415037	0.891	4.615169	0.24	1.588015	0.793	5.224111	0.298	2.682919	0.1
	men.	0.941784	0.84	4.101538	0.705	4.836016	0.378	1.580437	0.763	6.079295	0.883	2.979516	0.752
İzmir Konak Competition	1st	0.943378	0.46	3.72792	0.559	4.647542	0.398	1.648162	0.314	6.57004	1	2.997685	0.182
	2nd	0.971555	1	4.276124	0.905	5.219115	1	1.731529	0.745	5.186695	0.1	2.981762	0.1
	3rd	0.957413	0.729	4.426265	1	4.737869	0.493	1.606907	0.1	6.348176	0.856	2.986375	0.124
	men.	0.924609	0.1	3.169925	0.207	4.927702	0.693	1.780764	1	6.407956	0.895	3.013093	0.262
	men.	0.934703	0.294	3	0.1	4.471205	0.212	1.72301	0.703	5.539936	0.33	3.014446	0.269
	men	0.963035	0.837	3.934112	0.689	4.364418	0.1	1.663425	0.393	5.37593	0.223	3.155475	1
Bornova Competition	1st	0.963297	0.614	4.159871	1	4.969357	0.753	1.710713	0.412	5.619604	0.904	2.917254	0.328
	2nd	0.978192	0.859	1.678072	0.1	4.617947	0.1	1.89082	1	5.140538	0.635	3.12842	1
	3rd	0.981029	0.906	3.841302	0.884	5.102346	1	1.738566	0.503	5.282833	0.715	3.050947	0.754
	men.	0.97154	0.75	2.959358	0.565	4.749835	0.345	1.642048	0.188	5.791128	1	2.850221	0.115
	men.	0.986765	1	2.292782	0.323	4.965789	0.746	1.683515	0.323	5.643978	0.917	2.845477	0.1
	men	0.932019	0.1	3.066089	0.603	4.684298	0.223	1.615039	0.1	4.188083	0.1	2.974437	0.51
Efeler Competition	1st	0.938863	0.374	3.984893	0.537	5.333065	1	1.856088	1	6.162822	1	2.980546	0.372
	2nd	0.918106	0.1	4.297681	0.689	4.714441	0.687	1.855612	0.996	5.73429	0.632	2.970975	0.343
	3rd	0.986254	1	4.93546	1	4.870287	0.766	1.748326	0.135	5.787406	0.678	3.184799	1
	men.	0.955296	0.591	4.47032	0.773	3.556136	0.1	1.807419	0.609	5.113745	0.1	2.963359	0.319
	men.	0.979432	0.91	3.30117	0.204	4.358372	0.506	1.782523	0.409	5.273683	0.237	2.892056	0.1
	men	0.93938	0.381	3.087463	0.1	4.972297	0.817	1.743968	0.1	5.507764	0.438	2.972305	0.347
İnegöl Competition	1st	0.980802	0.739	4.89724	1	5.029065	0.746	1.700368	0.43	5.836829	0.863	2.900013	0.37
	2nd	0.94571	0.1	3.357552	0.1	4.938503	0.655	1.639837	0.193	5.939894	1	3.12049	0.754
	3rd	0.995143	1	4.689299	0.878	5.280644	1	1.615947	0.1	5.588391	0.534	2.744656	0.1
	men.	0.949712	0.173	4.169925	0.575	4.814403	0.53	1.8464	1	5.776155	0.783	3.261757	1
	men.	0.984337	0.803	3.906891	0.421	5.017908	0.735	1.78058	0.743	5.51408	0.436	2.947879	0.454
	men	0.957683	0.318	4.459432	0.744	4.38747	0.1	1.7638	0.677	5.260684	0.1	2.936011	0.433
Van İpekyolu Competition	1st	0.982175	0.877	3.643856	0.527	4.835777	1	1.733039	0.471	5.84786	1	3.117041	0.661
	2nd	0.991154	1	4.044394	0.792	4.779025	0.833	1.770905	0.783	5.770091	0.852	3.114834	0.658
	3rd	0.940938	0.315	4.357552	1	4.530063	0.1	1.797334	1	5.631685	0.589	3.206914	0.808
	men.	0.946242	0.387	3.369234	0.345	4.680503	0.543	1.737181	0.505	5.621775	0.57	3.324004	1
	men.	0.92519	0.1	3.882643	0.685	4.708856	0.626	1.749402	0.606	5.374546	0.1	2.774173	0.1
	men	0.958068	0.549	3	0.1	4.69623	0.589	1.687918	0.1	5.751156	0.816	2.971159	0.422
Süleymanpaşa Competition	1st	0.941682	0.1	3.922832	0.452	5.508047	1	1.788439	0.86	5.997753	1	2.787446	0.1
	2nd	0.965154	0.507	4.0703389	0.539	5.041382	0.539	1.781088	0.761	5.400189	0.265	3.03925	0.937
	3rd	0.993172	0.994	3.321928	0.1	5.06887	0.566	1.768058	0.58	5.325529	0.173	2.945325	0.625
	men.	0.993544	1	4.857981	1	5.410396	0.904	1.794857	0.952	5.969925	0.996	2.958712	0.669
	men.	0.964931	0.503	4.087463	0.549	4.597045	0.1	1.798316	1	5.60549	0.518	2.986426	0.761
	men	0.963333	0.476	3.459432	0.181	4.621018	0.124	1.733416	0.1	5.265942	0.1	3.058231	1

**Table 3:** Weight coefficient and Weighted Overall Entropy (WOE) calculations based on each single competition.

Competition	Status	Factor 1 Weight	Factor 1 Entropy	Factor 2 Weight	Factor 2 Entropy	Factor 3 Weight	Factor 3 Entropy	Factor 4 Weight	Factor 4 Entropy	Factor 5 Weight	Factor 5 Entropy	Factor 6 Weight	Factor 6 Entropy	Weighted Overall Entropy
Tekirdağ Competition	1st	3.191	0.806	1	0.1	1.15	0.1	3.754	0.825	4.897	0.268	4.996	1	12.192392
	2nd		0.822		1		0.887		0.899		1		0.455	15.188078
	3rd		0.993		0.442		1		1		0.985		0.25	14.632172
	men.		0.1		0.457		0.118		0.1		0.1		0.28	3.17578
	men.		1		0.891		0.24		0.793		0.298		0.1	9.293828
	men.		0.84		0.705		0.378		0.763		0.883		0.752	14.765485
İzmir Konak Competition	1st	2.209	0.46	3.773	0.559	4.178	0.398	1.145	0.314	3.036	1	1.878	0.182	8.525417
	2nd		1		0.905		1		0.745		0.1		0.1	11.14599
	3rd		0.729		1		0.493		0.1		0.856		0.124	10.389303
	men.		0.1		0.207		0.693		1		0.895		0.262	8.251521
	men.		0.294		0.1		0.212		0.703		0.33		0.269	4.224479
	men		0.837		0.689		0.1		0.393		0.223		1	7.871343
Bornova Competition	1st	1.601	0.614	4.208	1	1.163	0.753	4.364	0.412	3.477	0.904	4.802	0.328	12.582985
	2nd		0.859		0.1		0.1		1		0.635		1	13.286254
	3rd		0.906		0.884		1		0.503		0.715		0.754	14.635233
	men.		0.75		0.565		0.345		0.188		1		0.115	8.829167
	men.		1		0.323		0.746		0.323		0.917		0.1	8.905963
	men		0.1		0.603		0.223		0.1		0.1		0.51	6.631933
Efeler Competition	1st	4.746	0.374	1.894	0.537	2.137	1	4.789	1	2.569	1	3.962	0.372	13.760946
	2nd		0.1		0.689		0.687		0.996		0.632		0.343	11.000103
	3rd		1		1		0.766		0.135		0.678		1	14.627239
	men.		0.591		0.773		0.1		0.609		0.1		0.319	8.919927
	men.		0.91		0.204		0.506		0.409		0.237		0.1	8.750312
	men		0.381		0.1		0.817		0.1		0.438		0.347	6.722491
İnegöl Competition	1st	1.545	0.739	1.657	1	4.465	0.746	1.014	0.43	4.992	0.863	1.539	0.37	11.443191
	2nd		0.1		0.1		0.655		0.193		1		0.754	9.592883
	3rd		1		0.878		1		0.1		0.534		0.1	10.385874
	men.		0.173		0.575		0.53		1		0.783		1	10.048246
	men.		0.803		0.421		0.735		0.743		0.436		0.454	8.848627
	men		0.318		0.744		0.1		0.677		0.1		0.433	4.022683
Van İpekyolu Competition	1st	2.566	0.877	3.021	0.527	3.92	1	3.316	0.471	1.996	1	2.059	0.661	12.651284
	2nd		1		0.792		0.833		0.783		0.852		0.658	13.850274
	3rd		0.315		1		0.1		1		0.589		0.808	10.358936
	men.		0.387		0.345		0.543		0.505		0.57		1	9.018047
	men.		0.1		0.685		0.626		0.606		0.1		0.1	7.191901
	men		0.549		0.1		0.589		0.1		0.816		0.422	6.824468
Süleymanpaşa Competition	1st	3.955	0.1	1	0.452	4	1	1.806	0.86	4.369	1	3.627	0.1	11.13236
	2nd		0.507		0.539		0.539		0.761		0.265		0.937	10.630835
	3rd		0.994		0.1		0.566		0.58		0.173		0.625	10.365462
	men.		1		1		0.904		0.952		0.996		0.669	16.937229
	men.		0.503		0.549		0.1		1		0.518		0.761	9.767654
	men		0.476		0.181		0.124		0.1		0.1		1	6.80408

**Table 4:** Weight coefficient and WOE calculations based on multiple competitions.

Competition	Status	Factor 1 Weight	Factor 1 Entropy	Factor 2 Weight	Factor 2 Entropy	Factor 3 Weight	Factor 3 Entropy	Factor 4 Weight	Factor 4 Entropy	Factor 5 Weight	Factor 5 Entropy	Factor 6 Weight	Factor 6 Entropy	Weighted Overall Entropy
Tekirdağ Competition	1st	1.453	0.806	3.503	0.1	4.473	0.1	1.571	0.825	2.951	0.268	3.805	1	7.860661
	2nd		0.822		1		0.887		0.899		1		0.455	14.759521
	3rd		0.993		0.442		1		1		0.985		0.25	12.927385
	men.		0.1		0.457		0.118		0.1		0.1		0.28	3.791585
	men.		1		0.891		0.24		0.793		0.298		0.1	8.153394
	men.		0.84		0.705		0.378		0.763		0.883		0.752	12.046695
İzmir Konak Competition	1st		0.46		0.559		0.398		0.314		1		0.182	8.543615
	2nd		1		0.905		1		0.745		0.1		0.1	1.094221
	3rd		0.729		1		0.493		0.1		0.856		0.124	9.922402
	men.		0.1		0.207		0.693		1		0.895		0.262	9.179265
	men.		0.294		0.1		0.212		0.703		0.33		0.269	4.827546
	men		0.837		0.689		0.1		0.393		0.223		1	9.157504
Bornova Competition	1st		0.614		1		0.753		0.412		0.904		0.328	12.326307
	2nd		0.859		0.1		0.1		1		0.635		1	9.295612
	3rd		0.906		0.884		1		0.503		0.715		0.754	14.655218
	men.		0.75		0.565		0.345		0.188		1		0.115	8.296053
	men.		1		0.323		0.746		0.323		0.917		0.1	9.515327
	men		0.1		0.603		0.223		0.1		0.1		0.51	5.647838
Efeler Competition	1st		0.374		0.537		1		1		1		0.372	12.834993
	2nd		0.1		0.689		0.687		0.996		0.632		0.343	10.366681
	3rd		1		1		0.766		0.135		0.678		1	14.400181
	men.		0.591		0.773		0.1		0.609		0.1		0.319	6.479476
	men.		0.91		0.204		0.506		0.409		0.237		0.1	6.022606
	men		0.381		0.1		0.817		0.1		0.438		0.347	7.328307
İnegöl Competition	1st		0.739		1		0.746		0.43		0.863		0.37	12.543718
	2nd		0.1		0.1		0.655		0.193		1		0.754	9.548588
	3rd		1		0.878		1		0.1		0.534		0.1	11.115068
	men.		0.173		0.575		0.53		1		0.783		1	12.322917
	men.		0.803		0.421		0.735		0.743		0.436		0.454	10.110536
	men		0.318		0.744		0.1		0.677		0.1		0.433	6.521818
Van İpekyolu Competition	1st		0.877		0.527		1		0.471		1		0.661	13.799408
	2nd		1		0.792		0.833		0.783		0.852		0.658	1.420142
	3rd		0.315		1		0.1		1		0.589		0.808	10.791574
	men.		0.387		0.345		0.543		0.505		0.57		1	1.048011
	men.		0.1		0.685		0.626		0.606		0.1		0.1	6.972579
	men		0.549		0.1		0.589		0.1		0.816		0.422	7.95342
Süleymanpaşa Competition	1st		0.1		0.452		1		0.86		1		0.1	10.884216
	2nd		0.507		0.539		0.539		0.761		0.265		0.937	10.578566
	3rd		0.994		0.1		0.566		0.58		0.173		0.625	8.126128
	men.		1		1		0.904		0.952		0.996		0.669	15.891395
	men.		0.503		0.549		0.1		1		0.518		0.761	9.096529
	men		0.476		0.181		0.124		0.1		0.1		1	6.137523
